# Treatment provision and management for the menopause: a multinational survey study

**DOI:** 10.3389/fgwh.2025.1638428

**Published:** 2025-11-03

**Authors:** Nayra A. Martin-Key, Erin L. Funnell, Sabine Bahn

**Affiliations:** Department of Chemical Engineering and Biotechnology, Cambridge Centre for Neuropsychiatric Research, University of Cambridge, Cambridge, United Kingdom

**Keywords:** menopause, patient-centered care, health services accessibility, patient satisfaction, menopausal treatment, healthcare experiences, healthcare interactions

## Abstract

**Introduction:**

Despite available safe hormonal and non-hormonal interventions, most women with troublesome menopausal symptoms do not receive effective, evidence-based therapy, with notable international disparities in provision. This study aimed to investigate self-reported menopausal care experiences in a self-selecting sample from five English-speaking countries: Australia, Canada, New Zealand, the United Kingdom, and the United States, through an anonymous online survey.

**Methods:**

The 15–20 min survey, delivered via Qualtrics XM®, included questions on sociodemographic characteristics and treatment experiences, such as the number of healthcare professionals (HCP) seen before getting a prescription, ease of obtaining treatment, involvement in treatment discussions, appropriateness of treatment review and optimization, side effect tolerability, and overall satisfaction.

**Results:**

Data from 3,062 respondents were analyzed: Australia (16.59%, *n* = 508), Canada (17.54%, *n* = 537), New Zealand (16.59%, *n* = 508), UK (24.00%, *n* = 735), and US (25.28%, *n* = 774). Significant international differences were observed in both healthcare access and prescribing patterns. More women in the UK and US consulted an HCP compared with Australia, Canada, and New Zealand [*χ*²(4, *N* = 3062) = 101.02, *p* < 0.001, *φ*c = 0.18]. Prescription rates were higher in New Zealand, the UK, and the US compared with Australia and Canada [*χ*²(4, *N* = 2,485) = 75.71, *p* < 0.001, *φ*c = 0.18]. However, UK respondents, despite longer treatment use, generally reported less involvement in treatment discussions, poorer treatment review, lower side effect tolerability, and reduced satisfaction compared with other countries across treatment types.

**Discussion:**

Based on a self-selected cohort, these findings reveal critical gaps in menopause care, including disparities in treatment access and international differences in patient involvement. Greater access to healthcare in the UK and the US did not translate into higher satisfaction, highlighting the need for patient-centered approaches. Improving care requires better clinician education and strategies to enhance communication and shared decision-making.

## 1 Introduction

The menopause signifies the permanent end of menstrual cycles. It usually happens naturally between the ages of 44 and 55 years (1) due to a decline in ovarian follicular activity (2). However, the menopause can occur earlier for example if induced by surgical procedures, medication, or severe illness (3). The perimenopause, or menopause transition, is the phase leading up to natural menopause and is characterized by reduced ovarian function and irregular menstrual cycles. The post-menopause is the stage after menopause, defined by the absence of menstrual cycles for over a year, and women typically spend around 40% of their lives in this phase.

Critically, the symptoms associated with the menopause and its transition can present substantial challenges. Up to 80% of women experience difficulties during this period, with 25% rating these challenges as severe (4). Both the perimenopause and post-menopause stages are often accompanied by vasomotor symptoms (e.g., hot flashes and night sweats), physical symptoms (e.g., fatigue and bone and joint pain), and sexual symptoms (e.g., loss of libido and vaginal dryness during intercourse), all of which can significantly impact an individual's quality of life (5). Furthermore, menopause, particularly during the transition phase, can increase the risk of mental health issues (6), including depression, anxiety, and even suicidal thoughts (7–10).

Evidence suggests that the majority of women experiencing troublesome menopausal symptoms do not receive approved evidence-based therapy effectively (11, 12). In particular, it seems that women entering the early stages of their menopausal transition are less likely to receive treatment and support compared to those in the post-menopausal stage (13). Previous studies have revealed that many women perceive their doctors as being overly cautious when it comes to prescribing hormone-based treatments (13, 14). This perception is likely influenced by nearly two decades of widespread, conflicting, and often alarming information about menopause treatments that has been disseminated to both healthcare providers and the general public. The situation is further complicated by the lack of menopause assessment and treatment training in many undergraduate and postgraduate medical education programs (15).

Furthermore, there are considerable disparities in the availability and licensing of menopause treatment and support options worldwide. For instance, while female-specific testosterone therapy is readily available for the treatment of sexual symptoms associated with the menopause in countries such as Australia (16), this is not the case for the UK (unless prescribed privately), where a fractionated dose of an approved male formulation may be prescribed off-licence (17).

Even within countries, women often encounter a “postcode lottery” for menopausal care, leading to disparities in access and quality (18, 19). However, there is limited data on women's experiences and preferences regarding menopausal treatment and support, both within and across countries. Gaps in the data make it difficult to systematically understand how women navigate support across the perimenopause and post-menopause.

Therefore, this cross-sectional, exploratory, descriptive study aimed to investigate self-reported experiences with menopausal treatment and support options in five English-speaking countries: Australia, Canada, New Zealand, the United Kingdom (UK), and the United States (US). The primary outcomes of interest included the number of healthcare professionals consulted before receiving a prescription, perceived ease of obtaining treatment, involvement in treatment discussions, appropriateness of treatment review and optimization, side effect tolerability, and overall satisfaction. Each of these outcomes will be explored for each menopause treatment.

The outcomes of this study hold considerable implications for shaping future healthcare policies and guidelines, as well as enhancing the overall quality of care for women struggling with menopausal symptoms.

## 2 Methods

### 2.1 Study design

An online survey was developed using Qualtrics XM® and made available in five English-speaking countries.

### 2.2 Recruitment

Participants were recruited between December 2023 and February 2024 via email, paid Facebook and Instagram advertisements, free posts on the Cambridge Centre for Neuropsychiatric Research Facebook and X (formerly known as Twitter) pages, and Reddit. Recruitment messages were also disseminated by word-of-mouth and through relevant foundations and support groups. Recruitment materials emphasized that the focus of the study was on experiences of provision (treatment and support options) for the menopause.

### 2.3 Participants

Inclusion criteria for the study were: (1) ≥18 years, (2) assigned female at birth, (3) strong comprehension of the English language, and (4) currently experiencing symptoms of the menopause or menopause transition (e.g., hot flushes, mood changes, night sweats, irregular or absent periods, decreased sex drive). Exclusion criteria included current pregnancy, breastfeeding, or having given birth within the past year. Participants were required to confirm they were eligible based on this inclusion and exclusion criteria before consenting to participate in the study. Participants were *not* required to have sought help from a HCP or used any menopause treatments to take part.

### 2.4 Materials

As no existing validated questionnaire comprehensively captured the range of topics relevant to our outcomes of interest (i.e., treatment access, treatment satisfaction) the research team designed a bespoke survey to explore self-reported experiences of menopause care across multiple countries. The survey questions and accompanying study materials were designed in consultation with an experienced psychiatrist (SB). Technical piloting (i.e., end to end testing of the survey) was completed by two authors (NMK, EF). The online survey could be completed in 15–20 min and comprised eight sections: (1) participant information sheet detailing the rationale for the study, (2) electronic consent form, (3) socio-demographic information, (4) healthcare and treatment provision for the menopause [including: transdermal hormone replacement therapy (HRT), oral HRT, vaginal HRT, antidepressants, testosterone, cognitive behavioral therapy (CBT)/other type of therapy or counselling] (5) lifestyle changes and non-prescription medicines or supplements, (6) perceived usefulness of treatment and/or support regime, and positive and negative aspects of healthcare experiences, and (7) debrief. Section 6 comprised open-ended questions regarding which combination of treatment and/or support options for the menopause respondents found the most useful, as well as any other aspects or sources of help that have been useful, and positive and negative experiences for menopausal symptoms by HCPs.

For the purpose of the current analysis, only data from sections 3 and 4 were included as these specifically focused on participants’ experiences with menopause-related healthcare and treatment provision. This decision was made to keep the scope of the paper narrow and concise, allowing for a focused analysis. The remaining sections covered broader topics outside the aims of this study and will be analyzed in future manuscripts. The questions in section 4 were about experiences with treatment and support options for the menopause, including the number of different HCPs they had to see before being prescribed a particular treatment, the ease of being prescribed a particular treatment, the extent to which they felt involved in discussing treatment options, the extent to which they felt their treatment had been appropriately reviewed and optimised, the tolerability of side effects, and overall satisfaction levels. The survey was adaptive in nature, such that only relevant questions were asked based on previous responses.

### 2.5 Data analytic strategy

Data were processed and analyzed in SPSS version 28.0.1.1. Group differences (i.e., country: Australia, Canada, New Zealand, UK, US) in continuous variables were explored using one-factor ANOVAs, with effect sizes reported as eta-squared (*η*^2^; small = 0.01, medium = 0.06, large = 0.14) (20). Where appropriate, pairwise comparisons subject to the Bonferroni-correction method for multiple comparisons were conducted. Group differences in ordinal variables were explored using Kruskal–Wallis *H* tests, with posthoc Mann–Whitney *U*-tests subject to the Bonferroni-correction method for multiple comparisons conducted where appropriate. Effect sizes are reported as *r* (small = 0.10, medium = 0.30, large = 0.50) (20). Comparisons on binary variables were conducted using Chi-Square tests (*χ²*) or Fisher's Exact Test (FET) for low frequency data (i.e., values below five). Effect sizes are reported as Cramer's V (*φ*c; small = 0.10, medium = 0.30, large = 0.50) (21).

### 2.6 Ethical approval and informed consent

The study was approved by the University of Cambridge Psychology Research Ethics Committee (approval number PRE.2023.123). All participants provided informed consent electronically to participate in the study before commencing the survey.

## 3 Results

### 3.1 Sociodemographic characteristics

Respondents’ sociodemographic information per country, with between-group comparisons, can be found in Supplementary Table 1. Data from a total of 3,062 respondents who had completed at least 88% of the survey were included for analysis: Australia (16.59%, *n* = 508), Canada (17.54%, *n* = 537), New Zealand (16.59%, *n* = 508), UK (24.00%, *n* = 735), US (25.28%, *n* = 774).

### 3.2 Healthcare and treatment provision for the menopause

For an overview of healthcare and treatment provision for the menopause per country, with between-group comparisons, see Supplementary Table 2. There was a significant between-group difference in the proportion of respondents who had seen a HCP for menopausal symptoms [*χ²*(4, *N* = 3,062) = 101.02, *p* < 0.001, *φ*c = 0.18], with a significantly higher proportion of respondents in both the UK (88.40, *n* = 613) and the US (91.73%, *n* = 710) having visited a HCP for the menopause relative to those in Australia (74.41%, *n* = 378), Canada (73.74%, *n* = 396), and New Zealand (76.38%, *n* = 388). A significantly higher proportion of respondents in the US had seen a HCP for the menopause in comparison to those in the UK.

**Figure 1:**
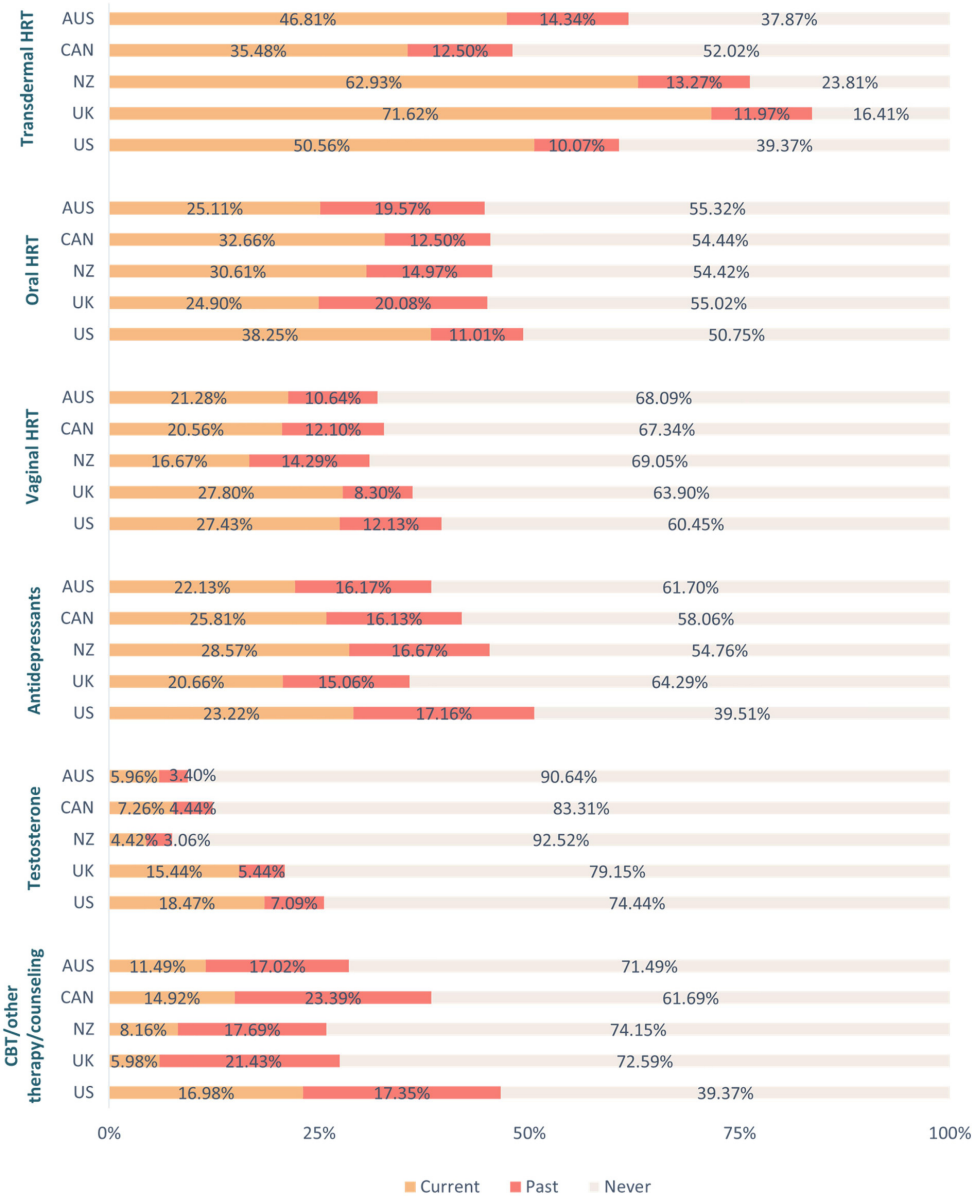
Use of treatment and support options for the menopause per country. *Key.* AUS, Australia; CAN, Canada; CBT, cognitive behavioral therapy; HRT, hormone replacement therapy; NZ, New Zealand; UK, United Kingdom; US, United States.

**Figure 2:**
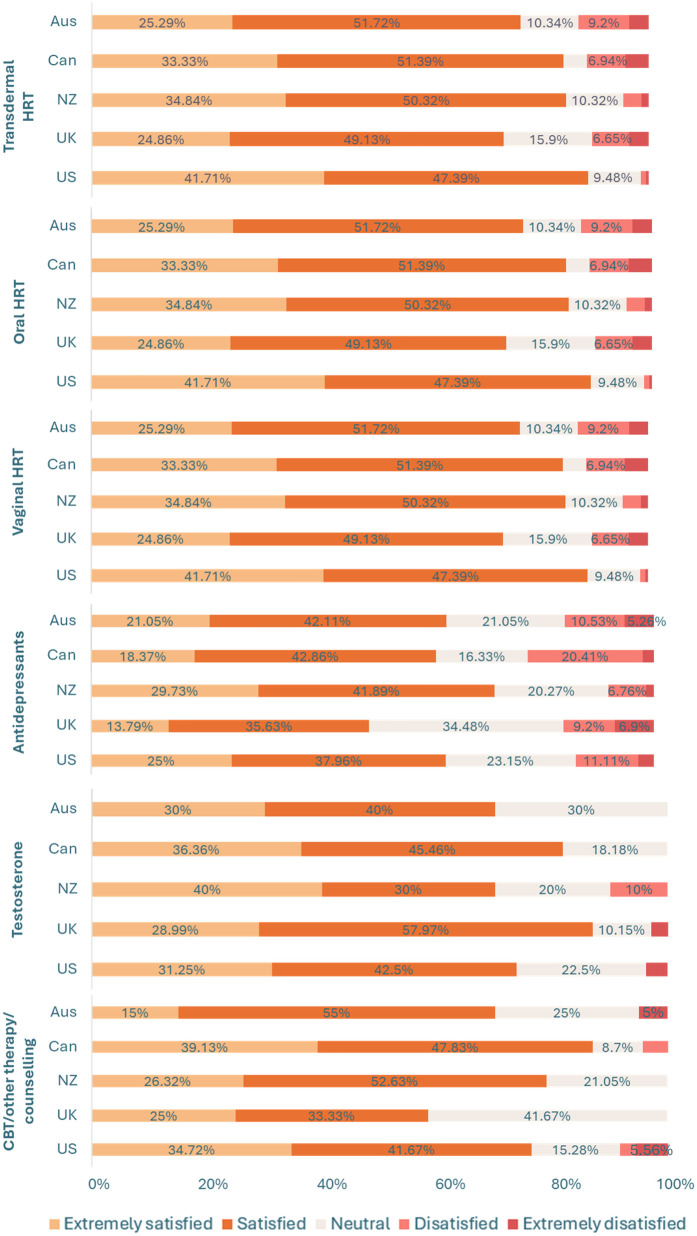
Overall satisfaction with different menopausal treatment types between countries. Data labels for percentages <5% have been removed for readability, see Supplementary Table 3. *Key.* AUS, Australia; CAN, Canada; CBT, cognitive behavioral therapy; HRT, hormone replacement therapy; NZ, New Zealand; UK, United Kingdom; US, United States.

Similarly, there was a significant between-group difference in the proportion of respondents who had been prescribed treatment and/or support options for the menopause [*χ²*(4, *N* = 2485) = 75.71, *p* < 0.001, *φ*c = 0.18]. A higher proportion of respondents in New Zealand (77.58%, *n* = 301), the UK (85.81%, *n* = 526), and the US (79.58%, *n* = 565) had been prescribed treatment and/or support options for the menopause relative to those in Australia (66.40%, *n* = 251) and Canada (66.92%, *n* = 265). A significantly higher proportion of those in the UK had been prescribed treatment and/or support options for the menopause in comparison to respondents in both New Zealand and the US. For an overview of treatment use per country see Figure 1, as well as Supplementary Table 2. For an overview of overall satisfaction per menopause treatment across countries see Figure 2, as well as Supplementary Table 3.

#### 3.2.1 Transdermal HRT

For an overview of current use of transdermal HRT per country, with between-group comparisons, see Supplementary Table 3. There was a significant group difference in the number of different HCPs respondents saw before being prescribed transdermal HRT [*H*(4) = 12.33, *p* = 0.02], with respondents in New Zealand seeing fewer different HCPs than respondents in both the UK (*U* = 10,633, *p* = 0.05, *r* = 0.15) and the US (*U* = 7,179, *p* = 0.02, *r* = 0.19). There was also a significant group difference in length of use, with respondents in the UK having used transdermal HRT for a longer duration than respondents in all other countries [*H*(4) = 67.72, *p* < 0.001; *Us* ≥ 11,590, *ps* = 0.01, *rs* ≥ 0.15]. A significant group difference was also found for the extent to which respondents felt they had been involved in discussing transdermal HRT [*H*(4) = 32.36, *p* < 0.001], with respondents in the UK feeling less involved than respondents in both Australia (*U* = 11,988.50, *p* = 0.02, *r* = 0.15) and the US (*U* = 26,971, *p* = 0.01, *r* = 0.23).

Similarly, a significant group difference emerged for the extent to which respondents felt their transdermal HRT had been appropriately reviewed by their HCP [*H*(4) = 55.57, *p* < 0.001], with respondents in the UK feeling less positive than respondents in all other countries (*Us* ≥ 9,205, *ps* = 0.01, *rs* ≥ 0.17). Related to this, a significant group difference was found for treatment optimisation [*H*(4) = 22.09, *p* < 0.001], with respondents in the UK feeling that their transdermal HRT treatment had not been as well optimised as respondents in Australia, New Zealand, and the US (*Us* ≥ 12,270, *ps* ≤ 0.05, *rs* ≥ 0.13). There was also a group difference in tolerability of side effects [*H*(4) = 40.35, *p* < 0.001], with respondents in Australia being less satisfied than those in the US (*U* = 6,901.50, *p* = 0.01, *r* = 0.21), and those in the UK being less satisfied relative to respondents in Canada, New Zealand, and the US (*Us* ≥ 9,401, *ps* ≤ 0.05, *rs* ≥ 0.13). Furthermore, a significant group difference was found for overall treatment satisfaction [*H*(4) = 32.36, *p* < 0.001]. Respondents in Australia reported lower levels of overall satisfaction with their transdermal HRT than those in the US (*U* = 7,093.50, *p* = 0.01, *r* = 0.20), and those in the UK were less satisfied with their transdermal HRT relative to respondents in both New Zealand and the US (*Us* ≥ 22,488, *ps* ≤ 0.02, *rs* ≥ 0.14).

#### 3.2.2 Oral HRT

For an overview of current use of oral HRT per country, with between-group comparisons, see Supplementary Table 3. There was a significant group difference in the number of different HCPs respondents had seen before being prescribed oral HRT [*H*(4) = 16.63, *p* = 0.002], with respondents in Australia and New Zealand having seen fewer HCPs before being prescribed this treatment option relative to those in the US (*Us* ≥ 1,649, *ps* ≤ 0.02, *rs* ≥ 0.24). There was also a significant group difference in length of use [*H*(4) = 10.43, *p* = 0.03], with respondents in the UK having used this treatment option for a longer duration than those in the US (*U* = 10,477, *p* = 0.02, *r* = 0.17). A significant group difference also emerged for the extent to which respondents felt they had been involved in discussing this treatment option [*H*(4) = 24.57, *p* < 0.001], with respondents in the UK feeling less involved than those in Canada and the US (*Us* ≥ 2,739, *ps* ≤ 0.02, *rs* ≥ 0.21), as well as respondents in New Zealand feeling less involved than those in the US (*U* = 4,790, *p* = 0.01, *r* = 0.21). There was a significant group difference in the extent to which respondents felt their oral HRT had been appropriately reviewed by their HCP [*H*(4) = 2,962, *p* < 0.001]. Respondents in the UK expressed that this treatment option had been reviewed to a lesser extent in comparison to respondents from all other countries (*Us* ≥ 1,876, *ps* ≤ 0.03, *rs* ≥ 0.22).

Related to this, a significant group difference was found for treatment optimisation [*H*(4) = 22.70, *p* < 0.001], with respondents in the UK feeling that their oral HRT treatment had not been as well optimised as respondents in Canada, New Zealand, and the US (*Us* ≥ 2,811.50, *ps* ≤ 0.04, *rs* ≥ 0.22). There was also a group difference in tolerability of side effects [*H*(4) = 23.06, *p* < 0.001], with respondents in the UK being less satisfied relative to respondents in Canada, New Zealand, and the US (*Us* ≥ 2,649.50, *ps* = 0.01, *rs* ≥ 0.24). Furthermore, a significant group difference was found for overall treatment satisfaction [*H*(4) = 18.74, *p* < 0.001]. Respondents in the UK reported lower levels of overall satisfaction with their oral HRT than respondents in both Canada and the US (*Us* ≥ 2,781, *ps* ≤ 0.03, *rs* ≥ 0.22).

#### 3.2.3 Vaginal HRT

For an overview of current use of vaginal HRT per country, with between-group comparisons, see Supplementary Table 3. A significant group difference was found in the extent respondents felt their vaginal HRT had been appropriately reviewed [*H*(4) = 12.89, *p* = 0.01], with those in the UK feeling less positive than those in the US (*U* = 5,224, *p* = 0.01, *r* = 0.21). There was also a significant group difference in the tolerability of side effects [*H*(4) = 11.91, *p* = 0.02], with respondents in New Zealand expressing lower tolerability levels relative to those in the US (*U* = 1,334.50, *p* = 0.01, *r* = 0.28).

#### 3.2.4 Antidepressants

For an overview of current use of antidepressants for the menopause per country, with between-group comparisons, see Supplementary Table 3. A significant group difference emerged for ease of prescription of antidepressants for the menopause [*H*(4) = 10.60, *p* = 0.031]. Respondents in New Zealand expressed more difficulties being prescribed this treatment option relative to those in the US (*U* = 611.50, *p* = 0.05, *r* = 0.30). A significant group difference was also found for the extent to which respondents felt their antidepressants had been appropriately reviewed [*H*(4) = 26.17, *p* < 0.001], with those in the UK feeling less positive in comparison to respondents in all other countries (*Us* ≥ 1,030.50, *ps* ≤ 0.03, *rs* ≥ 0.23). Furthermore, a significant group difference was found for overall treatment satisfaction [*H*(4) = 10.67, *p* = 0.03]. Respondents in the UK reported lower levels of overall satisfaction with their antidepressants than respondents in New Zealand (*U* = 2,323.50, *p* = 0.01, *r* = 0.25).

#### 3.2.5 Testosterone

For an overview of current use of testosterone for the menopause per country, with between-group comparisons, see Supplementary Table 3. There was a significant group difference in length of treatment use [*H*(4) = 9.74, *p* = 0.05] but no group differences survived the Bonferroni-correction method for multiple comparisons. A significant group difference also emerged for the extent to which respondents felt their testosterone medication had been appropriately reviewed [*H*(4) = 11.92, *p* = 0.02], with those in the UK feeling less positive than respondents in the US (*U* = 2,024, *p* = 0.01, *r* = 0.27).

#### 3.2.6 CBT/other type of therapy or counselling

For an overview of current use of CBT/other type of therapy or counselling for the menopause per country, with between-group comparisons, see Supplementary Table 3. There was a significant group difference in length of therapy [*H*(4) = 25.97, *p* < 0.001], with respondents in the UK reporting shorter duration of therapy compared to those in Australia, New Zealand, and the US (*Us* ≥ 138, *ps* = 0.01, *rs* ≥ 0.42).

## 4 Discussion

Significant international differences were found in the provision and management of menopause-related treatment options; however, some of these differences had small effect sizes, suggesting that while statistically significant, it is not clear whether these differences would translate into meaningful variations in real-world clinical care or patient outcomes. Further, whilst the study identifies international differences in menopause care experiences, it cannot determine the underlying causes of these differences due to the cross-sectional study design. For example, the lower satisfaction rates for menopause reported in the UK may reflect genuine differences in care quality but also other unmeasured confounders such as cultural variation in reporting satisfaction with care or more systematic differences in care delivery between nations. More respondents in the UK and US sought medical advice for the menopause compared to their counterparts in Australia, Canada, and New Zealand. Moreover, a higher proportion of respondents in the UK, US, and New Zealand were prescribed treatment or support options for menopause compared to those in Australia and Canada. These findings may be indicative of a more proactive approach to medical management of menopausal symptoms and advocating for treatment in the UK and US, and to a lesser extent, in New Zealand. Indeed, in the UK, increased public awareness and recent media coverage of menopause-related issues may have shaped expectations of care, leading to a more proactive approach to engaging with healthcare, including making active requests for formal interventions. In fact, recent research conducted in the UK general population has revealed that women feel they need to take personal responsibility for their menopausal care, often resulting in having to conduct their own research and advocating for treatments like HRT (13). Notably, while self-advocacy can help empower and facilitate patient-doctor interactions, it should not be a prerequisite for quality care.

Relative to those in New Zealand, respondents in both the UK and the US had to see a higher number of different HCPs before being prescribed transdermal HRT, which may reflect variations in healthcare systems, accessibility, and prescribing practices. Notably, respondents in the UK reported longer durations of transdermal HRT use compared to other countries but expressed feeling less involved in treatment decisions. This is in line with extant research demonstrating that women in the UK often feel they are not adequately involved in decision-making processes or provided with sufficient information about treatment options for the menopause (13). Additionally, UK respondents perceived their transdermal HRT as less thoroughly reviewed and optimized. Respondents in both the UK and Australia reported lower tolerability of side effects. These findings highlight possible shortcomings in the provision and management of transdermal HRT, particularly in the UK.

Respondents from Australia and New Zealand reported obtaining an oral HRT prescription after seeing fewer HCPs compared to participants from other countries with New Zealanders also feeling less involved in treatment decisions. Despite longer use of oral HRT, UK respondents felt less involved in decisions, had lower treatment satisfaction, and found side effects less tolerable. They were also less satisfied with the review and optimization of their oral HRT, vaginal HRT, testosterone, and antidepressants compared to other countries. It is worth considering that these international differences in perceived optimization of treatment may reflect variation in primary care appointment lengths, with the UK offering shorter consultations than Australia and New Zealand (20). These shorter consultations may restrict the depth of discussion around menopause symptoms and treatment options, contributing to lower perceived involvement in decision-making and treatment review. New Zealanders faced more difficulty obtaining antidepressant prescriptions for the menopause and reported lower tolerability of vaginal HRT side effects. Additionally, UK respondents had shorter therapy or counseling durations than those in Australia, New Zealand, and the US. This likely reflects the limited number of sessions for psychological therapies (i.e., six sessions) available in a single referral through the publicly funded national health service in the UK.

Taken together, these findings highlight the urgent need to improve communication and patient involvement in menopause care, particularly in the UK. Many women report feeling unsupported and confused about symptom management, often attributing this to a lack of engagement from HCPs. At the same time, HCPs frequently feel underprepared to offer effective guidance (15), pointing to gaps in training and education. Policy efforts should prioritize integrating menopause education into medical curricula, offering ongoing professional development, and establishing evidence-based clinical guidelines. In parallel, healthcare systems must support the creation of dedicated menopause services, promote patient-centered communication, and encourage interprofessional collaboration. Embedding these strategies into national health policies will help build a more informed, responsive, and empathetic system of care that better serves the diverse needs of women during menopause.

This study has limitations that warrant discussion. The findings may not fully represent broader national populations, especially ethnic minorities and disadvantaged groups who often face greater barriers in menopausal care. Recruitment via social media and online surveys introduces bias, potentially over-representing those with negative experiences or a particular interest in menopause. These factors should be considered when interpreting the results. The study focused on high-income countries with better healthcare access than low- and middle-income countries (LMICs) (22, 23), where menopausal care faces additional challenges. Future research should explore these contexts. Despite these limitations, this study contributes important patient-centered insights to the under-researched area of menopausal care, highlighting key gaps in care and provider communication, while offering a valuable foundation for future research and policy development.

### 4.1 Conclusion

This study highlights international variations in self-reported menopause care within a self-selected sample. While not generalizable to all women, the findings underscore the importance of enhanced communication, increased patient involvement, and compassionate, individualized care. Empowering women through shared decision-making and tailored treatment can enhance outcomes. Establishing dedicated menopause clinics, support groups, and specialized services would help create a more comprehensive care environment. Integrating menopause education into medical training, ongoing professional development, and national guidelines is essential for equipping healthcare providers to manage symptoms effectively.

## Data Availability

The datasets presented in this article are not readily available because they are part of ongoing research. Requests to access the datasets should be directed to Prof Sabine Bahn, sb209@cam.ac.uk.
