# Supplementary material for: Treatment provision and management for the menopause: a multinational survey study
Source: Front Glob Womens Health. 2025 Nov 3;6:1638428. doi: 10.3389/fgwh.2025.1638428 (PMC12620394; doi:10.3389/fgwh.2025.1638428)
Supplement: Supplementary file 1 [file Table1.docx]

**Supplementary Materials**

**Supplementary Table 1.** Sociodemographic characteristics per country, with between-group comparisons (*N*=3,062)

|  |  | **Australia (*n*=508)** | **Canada (*n*=537)** | **New Zealand (*n*=508)** | **UK (*n*=735)** | **US (*n*=774)** |  |  |
| --- | --- | --- | --- | --- | --- | --- | --- | --- |
|  |  | ***M*(SD)** | | | | | ***p*** | **Post-hoc comparisons** |
| Age |  | 51.87  (5.07) | 52.15  (5.02) | 51.86  (4.90) | 51.58 (4.95) | 52.34 (5.92) | 0.06 | - |
|  |  |  |  | ***n* (%)** |  |  |  |  |
| Gender | Woman | 506  (99.61) | 532  (99.07) | 501  (98.62) | 722 (98.23) | 763 (98.58) | 0.76 | - |
|  | Man | 0 (0.00) | 0 (0.00) | 2 (0.39) | 2 (0.27) | 1 (0.13) |  |  |
|  | Non-binary | 2 (0.39) | 4 (0.74) | 3 (0.59) | 6 (0.82) | 5 (0.65) |  |  |
|  | Other | 0 (0.00) | 0 (0.00) | 0 (0.00) | 1 (0.14) | 2 (0.26) |  |  |
|  | Prefer not to answer | 0 (0.00) | 1 (0.19) | 2 (0.39) | 4 (0.54) | 3 (0.39) |  |  |
| Ethnicity | White/Caucasian | 466  (91.73) | 484  (90.13) | 425  (83.66) | 693 (94.29) | 688 (88.89) | <0.001*** | AUS,CAN,UK>NZ; UK>US |
|  | Asian | 13 (2.56) | 14 (2.61) | 6 (1.18) | 6 (0.82) | 9 (1.16) |  | - |
|  | Black/Caribbean/African | 1 (0.20) | 2 (0.37) | 1 (0.20) | 7 (0.95) | 11 (1.42) |  | - |
|  | Arab/Middle Eastern/North African descent | 3 (0.59) | 2 (0.37) | 0 (0.00) | 0 (0.00) | 2 (0.26) |  | - |
|  | Hispanic/Latinx | 3 (0.59) | 3 (0.56) | 2 (0.39) | 1 (0.14) | 34 (4.39) |  | US>all others |
|  | Mixed/multiple | 13 (2.56) | 25 (4.66) | 47 (9.25) | 23 (3.13) | 25 (3.23) |  | NZ>all others |
|  | Other | 6 (1.18) | 3 (0.56) | 23 (4.53) | 1 (0.14) | 4 (0.52) |  | NZ>all others |
|  | I am not sure | 0 (0.00) | 1 (0.19) | 0 (0.00) | 0 (0.00) | 1 (0.13) |  | - |
|  | Prefer not to answer | 3 (0.59) | 3 (0.56) | 4 (0.79) | 4 (0.54) | 0 (0.00) |  | - |
| Education^a^ | ≤ Primary (up to 11 years) | 20 (3.94) | 15 (2.79) | 3 (0.59) | 7 (0.95) | 8 (1.03) | <0.001*** | US>all others |
|  | Lower secondary (up to 16 years) | 33  (6.50) | 34  (6.33) | 58  (11.42) | 78 (10.61) | 37  (4.78) |  |  |
|  | Upper secondary (up to 18 years) | 83  (16.34) | 76  (14.15) | 82  (16.14) | 150 (20.41) | 57  (7.36) |  |  |
|  | Undergraduate/college degree (i.e., Bachelors) | 179  (35.24) | 270 (50.28) | 200  (16.14) | 245 (33.33) | 313 (40.44) |  |  |
|  | Postgraduate degree (e.g., Masters, PhD) | 152  (29.92) | 95  (17.69) | 124  (24.41) | 199 (27.07) | 341 (44.06) |  |  |
|  | Other | 32 (6.30) | 28 (5.21) | 28 (5.51) | 45 (6.12) | 13 (1.68) |  |  |
|  | Prefer not to answer | 9 (1.77) | 19 (3.54) | 13 (2.56) | 11 (1.50) | 5 (0.65) |  |  |
| Employment^b^ | Full-time | 223  (43.90) | 297 (55.31) | 268  (52.76) | 335 (45.58) | 413 (53.36) | <0.001*** | AUS<CAN,NZ,US; CAN>UK; UK<US |
|  | Part-time | 146  (28.74) | 67  (12.48) | 101  (19.88) | 164 (22.31) | 96 (12.40) | <0.001*** | AUS>CAN,NZ,US; CAN<NZ,UK; UK>US |
|  | Self-employed | 41  (8.07) | 58  (10.80) | 70  (13.78) | 93 (12.65) | 107 (13.82) | 0.02* | AUS<NZ,US |
|  | Parental leave/caring responsibilities | 8 (1.58) | 4 (0.75) | 8 (1.58) | 16 (2.18) | 10 (1.29) | 0.30 | - |
|  | Homemaker | 36  (7.09) | 48  (8.94) | 43  (8.45) | 45  (6.12) | 96 (12.40) | <0.001*** | AUS,UK<US |
|  | Student | 11 (2.17) | 7 (1.34) | 5 (0.98) | 9 (1.22) | 8 (1.03) | 0.43 | - |
|  | Voluntary work | 14 (2.76) | 11 (2.05) | 14 (2.76) | 22 (2.99) | 25 (3.23) | 0.78 | - |
|  | Retired | 30  (5.91) | 46  (8.57) | 14  (2.76) | 45  (6.12) | 79 (10.21) | <0.001*** | CAN,US>NZ; UK<US |
|  | Unemployed | 20 (3.94) | 22 (4.10) | 20 (3.94) | 36 (4.90) | 14 (1.81) | 0.02** | UK>US |
|  | Prefer not to answer | 15 (2.95) | 19 (3.52) | 10 (1.97) | 20 (2.72) | 6 (0.78) | 0.01** | AUS,CAN,UK>US |

| ***Note.*** AUS, Australia; CAN, Canada; NZ, New Zealand; UK, United Kingdom; US, United States.  ***Key.*** **p*<0.05; ***p*<0.01; ****p*<0.001 ^a^ data treated as ordinal ^b^ percentages add to more than 100% as participants could select multiple options  **Supplementary Table 2.** Healthcare and treatment provision for the menopause per country, with between-group comparisons (*N*=1,831^a^) | | | | | | | | |
| --- | --- | --- | --- | --- | --- | --- | --- | --- |
|  |  | **Australia** | **Canada** | **New Zealand** | **UK** | **US** |  |  |
|  |  | ***n* (%)** | | | | | ***p***^b^ | **Post-hoc comparisons** |
| Transdermal HRT | Yes, currently using | 110 (46.81) | 88 (35.48) | 185 (62.93) | 371 (71.62) | 271 (50.56) | <0.001*** | AUS<NZ,UK; CAN<all others; NZ,UK>US |
|  | Yes, in the past | 36  (14.34) | 31 (12.50) | 39 (13.27) | 62 (11.97) | 54 (10.07) |  |  |
|  | No, never | 89  (37.87) | 129 (52.02) | 70 (23.81) | 85 (16.41) | 211 (39.37) |  |  |
| Oral HRT^c^ | Yes, currently using | 59  (25.11) | 81 (32.66) | 90 (30.61) | 129 (24.90) | 205 (38.25) | 0.63 | - |
|  | Yes, in the past | 46  (19.57) | 32 (12.50) | 44 (14.97) | 104 (20.08) | 59 (11.01) |  |  |
|  | No, never | 130 (55.32) | 135 (54.44) | 160 (54.42) | 285 (55.02) | 272 (50.75) |  |  |
| Vaginal HRT^d^ | Yes, currently using | 50  (21.28) | 51 (20.56) | 49 (16.67) | 144 (27.80) | 147 (27.43) | 0.07 | - |
|  | Yes, in the past | 25  (10.64) | 30 (12.10) | 42 (14.29) | 43  (8.30) | 65 (12.13) |  |  |
|  | No, never | 160 (68.09) | 167 (67.34) | 203 (69.05) | 331 (63.90) | 324 (60.45) |  |  |
| Antidepressants^e^ | Yes, currently using | 52  (22.13) | 64 (25.81) | 84 (28.57) | 107 (20.66) | 125 (23.32) | 0.07 | - |
|  | Yes, in the past | 38  (16.17) | 40 (16.13) | 49 (16.67) | 78 (15.06) | 92 (17.16) |  |  |
|  | No, never | 145 (61.70) | 144 (58.06) | 161 (54.76) | 333 (64.29) | 319 (59.51) |  |  |
| Testosterone^f^ | Yes, currently using | 14  (5.96) | 18  (7.26) | 13  (4.42) | 80 (15.44) | 99 (18.47) | <0.001*** | AUS,CAN,NZ<UK,US |
|  | Yes, in the past | 8  (3.40) | 11  (4.44) | 9  (3.06) | 28  (5.44) | 38  (7.09) |  |  |
|  | No, never | 213 (90.64) | 219 (88.31) | 272 (92.52) | 410 (79.15) | 399 (74.44) |  |  |
| CBT/therapy/counselling^g^ | Yes, currently using | 27  (11.49) | 37 (14.92) | 24  (8.16) | 31  (5.98) | 91 (16.98) | 0.002** | CAN>NZ,UK |
|  | Yes, in the past | 40  (17.02) | 58 (23.39) | 52 (17.69) | 111 (21.43) | 93 (17.35) |  |  |
|  | No, never | 168 (71.49) | 153 (61.69) | 218 (74.15) | 376 (72.59) | 352 (65.67) |  |  |

***Note.*** AUS, Australia; CAN, Canada; CBT, cognitive behavioural therapy; HRT, hormone replacement therapy; NZ, New Zealand; UK, United Kingdom; US, United States.

***Key.*** ***p*<0.01; ****p*<0.001
^a^ 75 respondents selected ‘other’ treatment and were therefore not included, while data from 2 respondents were removed as responses were changed to ‘never having had treatment for the menopause’ after examining open-ended responses
^b^ analyses combined past and current use as having used a particular treatment *vs.* never
^c^ 2 responses were changed from ‘past’ to ‘current use’ as open-ended responses suggested ongoing use
^d^ 1 response was changed from ‘past use’ to ‘never’ as open-ended response suggested never having used this treatment
^e^ 28 responses were changed from ‘past use’ to ‘never’ and 6 responses were changed from ‘current use’ to ‘never’ as open-ended responses suggested use for reasons other than the menopause
^f^ 1 response was changed from ‘past use’ to ‘never’ as open-ended response suggested not having used this treatment and 1 response was changed from ‘current’ to ‘past use’ as open-ended response suggested discontinuation
^g^ 31 responses were changed from ‘past use’ to ‘never’ and 1 response was changed from ‘current use’ to ‘never’ as open-ended responses suggested having used this treatment for reasons other than the menopause, an additional 4 responses were changed from ‘past’ to ‘current use’ as open-ended responses suggested ongoing use

| **Supplementary Table 3.** Current treatment and support options for the menopause per country, with between-group comparisons | | | | | | | | |
| --- | --- | --- | --- | --- | --- | --- | --- | --- |
|  |  | **Australia** | **Canada** | **New Zealand** | **UK** | **US** |  |  |
| ***Transdermal HRT*** |  | ***n* (%)** | | | | | ***p*** | **Post-hoc comparisons** |
| How long to be prescribed current transdermal HRT by HCP^a,b^ | At first contact | 36  (32.73) | 23 (26.14) | 72 (38.92) | 143 (38.54) | 111 (40.96) | 0.44 | - |
|  | 1 – 3 months | 21  (19.09) | 19 (21.59) | 29 (15.68) | 60 (16.17) | 38 (14.02) |  |  |
|  | 3 – 6 months | 10  (9.09) | 5 (17.05) | 13 (17.03) | 32 (8.63) | 18 (6.64) |  |  |
|  | 6 months – 1 year | 7  (6.36) | 15 (17.05) | 14 (7.57) | 34 (9.16) | 24 (8.86) |  |  |
|  | 1 – 2 years | 15  (13.64) | 9 (10.23) | 17 (9.19) | 46 (12.40) | 20 (7.38) |  |  |
|  | More than 2 years | 21  (19.09) | 17 (19.32) | 40 (21.62) | 56 (15.09) | 60 (22.14) |  |  |
| Number of different HCPs before being prescribed current transdermal HRT^a,c^ | 1 | 19  (25.68) | 19 (29.23) | 43 (38.05) | 58 (25.44) | 30 (18.75) | 0.02* | NZ<UK,US |
|  | 2 – 3 | 37  (50.00) | 34 (52.31) | 49 (43.36) | 101 (44.30) | 88 (55.00) |  |  |
|  | 3 – 4 | 8  (10.81) | 6  (9.23) | 10  (8.85) | 34 (14.91) | 20 (12.50) |  |  |
|  | 4 – 5 | 3  (4.05) | 4  (6.15) | 6  (5.31) | 18  (7.89) | 10  (6.25) |  |  |
|  | More than 5 | 7  (9.46) | 2  (3.08) | 5  (4.42) | 17  (7.46) | 12  (7.50) |  |  |
| Ease of being prescribed current transdermal HRT by HCP^a,b^ | Very difficult | 9  (8.18) | 10 (11.36) | 10  (5.41) | 39 (10.63) | 26  (9.59) | 0.25 | - |
|  | Difficult | 19  (17.27) | 19 (21.59) | 27 (14.59) | 72 (19.62) | 51 (18.82) |  |  |
|  | Neutral | 16  (14.55) | 11 (12.50) | 29 (15.68) | 46 (12.53) | 37 (13.65) |  |  |
|  | Easy | 34  (30.91) | 24 (27.27) | 58 (31.35) | 107 (29.16) | 72 (26.57) |  |  |
|  | Very easy | 32  (29.09) | 24 (27.27) | 61 (32.97) | 103 (28.07) | 85 (31.37) |  |  |
| Length of current transdermal HRT use^a,b^ | Less than 3 months | 23  (20.91) | 16 (18.18) | 30  (16.22) | 25  (6.74) | 60 (22.14) | <0.001*** | UK>all others |
|  | 3 – 6 months | 16  (14.55) | 18 (20.45) | 26  (14.05) | 32  (8.63) | 48 (17.71) |  |  |
|  | 6 months – 1 year | 17  (15.45) | 21 (23.86) | 43  (23.24) | 70 (18.87) | 65 (23.99) |  |  |
|  | 1 – 2 years | 26  (23.64) | 14 (15.91) | 51  (27.57) | 134 (36.12) | 59 (21.77) |  |  |
|  | More than 2 years | 28  (25.45) | 19 (21.59) | 35  (18.92) | 110 (29.65) | 39 (14.39) |  |  |
| Extent to which involved in discussing current transdermal HRT use with HCP^a,d^ | Not at all | 6  (6.90) | 3  (4.17) | 7  (4.52) | 22  (6.36) | 4  (1.90) | <0.001*** | UK<AUS,US |
|  | To a small extent | 8  (9.20) | 10 (13.89) | 28  (18.06) | 81 (23.41) | 27 (12.80) |  |  |
|  | To some extent | 15  (17.24) | 13 (18.06) | 31  (20.00) | 77 (22.25) | 35 (16.59) |  |  |
|  | To a moderate extent | 21  (24.14) | 21 (29.17) | 34  (21.94) | 66 (19.08) | 43 (20.38) |  |  |
|  | To a large extent | 37  (42.53) | 25 (34.72) | 55  (35.48) | 100 (28.90) | 102 (48.34) |  |  |
| Has current transdermal HRT been appropriately reviewed by HCP^a,d^ | Yes | 57  (65.52) | 43 (59.72) | 89  (57.42) | 127 (36.71) | 135 (63.98) | <0.001*** | UK<all others |
|  | No | 16  (18.39) | 10 (13.89) | 34  (21.94) | 102 (29.48) | 23 (10.90) |  |  |
|  | Mixed experience | 14  (16.09) | 19 (26.39) | 32  (20.65) | 117 (33.82) | 53 (25.12) |  |  |
| Has current transdermal HRT been appropriately optimised^a,d^ | Not at all | 10  (11.49) | 5  (6.94) | 16  (10.32) | 58 (16.76) | 23 (10.90) | <0.001*** | UK<AUS,NZ,US |
|  | Yes, partially | 21  (24.14) | 25 (34.72) | 42  (27.10) | 124 (35.84) | 53 (25.12) |  |  |
|  | Yes, mostly | 35  (40.23) | 31 (43.06) | 67  (43.23) | 111 (32.08) | 79 (37.44) |  |  |
|  | Yes, completely | 21  (24.14) | 11 (15.28) | 30  (19.35) | 53 (15.32) | 56 (26.54) |  |  |
| Satisfaction with tolerability of current transdermal HRT^a,d^ | Extremely dissatisfied | 4  (4.60) | 1  (1.39) | 4  (2.58) | 13  (3.76) | 1  (0.47) | <0.001*** | AUS<US; UK<CAN,NZ,US |
|  | Dissatisfied | 10  (11.49) | 2  (2.78) | 12  (7.74) | 36 (10.40) | 12  (5.69) |  |  |
|  | Neutral | 12  (13.79) | 6  (8.33) | 24  (15.48) | 67 (19.36) | 20  (9.48) |  |  |
|  | Satisfied | 35  (40.23) | 33 (45.83) | 50  (32.26) | 133 (38.44) | 73 (34.60) |  |  |
|  | Extremely satisfied | 26  (29.89) | 30 (41.67) | 65  (41.94) | 97 (28.03) | 105 (49.76) |  |  |
| Overall satisfaction with current transdermal HRT^a,d^ | Extremely dissatisfied | 3  (3.45) | 3  (4.17) | 2  (1.29) | 12  (3.47) | 1  (0.47) | <0.001*** | AUS<US; UK<NZ,US |
|  | Dissatisfied | 8  (9.20) | 5  (6.94) | 5  (3.23) | 23  (6.65) | 2  (0.95) |  |  |
|  | Neutral | 9  (10.34) | 3  (4.17) | 16  (10.32) | 55 (15.90) | 20  (9.48) |  |  |
|  | Satisfied | 45  (51.72) | 37 (51.39) | 78  (50.32) | 170 (49.13) | 100 (47.39) |  |  |
|  | Extremely satisfied | 22  (25.29) | 24 (33.33) | 54  (34.84) | 86 (24.86) | 88 (41.71) |  |  |
| How long to be prescribed current oral HRT by HCP^a,f^ | At first contact | 21  (35.59) | 21 (25.93) | 33  (36.67) | 55 (42.97) | 79 (38.73) | 0.30 | - |
|  | 1 – 3 months | 14  (23.73) | 20 (24.69) | 11  (12.22) | 22 (17.19) | 35 (17.16) |  |  |
|  | 3 – 6 months | 6  (10.17) | 8  (9.88) | 5  (12.22) | 10  (7.81) | 16  (7.84) |  |  |
|  | 6 months – 1 year | 8  (13.56) | 7  (8.64) | 9  (10.00) | 6  (4.69) | 8  (3.92) |  |  |
|  | 1 – 2 years | 1  (1.70) | 8  (9.88) | 10  (11.11) | 14 (10.94) | 20  (9.80) |  |  |
|  | More than 2 years | 9  (15.25) | 17 (20.99) | 22  (24.44) | 21 (16.41) | 46 (22.50) |  |  |
| Number of different HCPs before being prescribed current oral HRT^a,g^ | 1 | 19  (50.00) | 21 (35.00) | 28  (49.12) | 23 (31.51) | 24 (19.20) | 0.002** | AUS,NZ<US |
|  | 2 – 3 | 13  (34.21) | 27 (45.00) | 19  (33.33) | 28 (38.36) | 68 (54.40) |  |  |
|  | 3 – 4 | 2  (5.26) | 6  (10.00) | 3  (5.26) | 8  (10.96) | 16 (12.40) |  |  |
|  | 4 – 5 | 2  (5.26) | 3  (5.00) | 5  (8.77) | 9  (12.33) | 9  (7.20) |  |  |
|  | More than 5 | 2  (5.26) | 3  (5.00) | 2  (3.51) | 5  (6.85) | 8  (6.40) |  |  |
| Ease of being prescribed current oral HRT by HCP^a,f^ | Very difficult | 2  (3.39) | 10 (12.35) | 7  (7.79) | 8  (6.25) | 17  (8.33) | 0.21 | - |
|  | Difficult | 3  (5.09) | 13 (14.82) | 13  (14.44) | 19 (14.84) | 34 (16.67) |  |  |
|  | Neutral | 8  (13.56) | 12 (14.82) | 11  (12.22) | 18 (14.06) | 24 (11.77) |  |  |
|  | Easy | 22  (37.29) | 19 (23.46) | 34  (37.78) | 43 (33.59) | 51 (25.00) |  |  |
|  | Very easy | 24  (40.68) | 27 (33.33) | 25  (27.79) | 40 (31.25) | 78 (38.24) |  |  |
| Length of current oral HRT use^a,f^ | Less than 3 months | 12  (20.34) | 16 (19.75) | 12  (13.33) | 13 (10.16) | 39 (19.12) | 0.003** | UK>US |
|  | 3 – 6 months | 12  (20.34) | 15 (18.52) | 14 (15.560 | 13 (10.16) | 26 (12.75) |  |  |
|  | 6 months – 1 year | 6  (10.17) | 13 (16.05) | 25  (27.78) | 18 (14.06) | 44 (21.57) |  |  |
|  | 1 – 2 years | 10  (16.95) | 9  (11.11) | 23  (25.56) | 47 (36.72) | 53 (25.98) |  |  |
|  | More than 2 years | 19  (32.20) | 28 (34.57) | 16  (17.78) | 37 (28.91) | 42  (5.89) |  |  |
| Extent to which involved in discussing current oral HRT use with HCP^a,h^ | Not at all | 3  (6.38) | 2  (3.08) | 4  (5.13) | 12 (10.44) | 7  (4.24) | <0.001*** | CAN>UK; NZ,UK<US |
|  | To a small extent | 7  (14.89) | 10 (15.39) | 16  (20.51) | 34 (29.57) | 21 (12.73) |  |  |
|  | To some extent | 12  (25.53) | 13 (20.00) | 25  (32.05) | 20 (17.39) | 31 (18.79) |  |  |
|  | To a moderate extent | 12  (25.53) | 12 (18.46) | 16  (20.51) | 19 (16.52) | 34 (20.61) |  |  |
|  | To a large extent | 13  (27.66) | 28 (43.08) | 17  (21.80) | 30 (26.09) | 72 (43.64) |  |  |
| Has current oral HRT been appropriately reviewed by HCP^a,h^ | Yes | 28  (59.57) | 32 (49.23) | 43  (55.13) | 34 (29.57) | 92 (55.78) | <0.001*** | UK<all others |
|  | No | 9  (19.15) | 11 (16.92) | 17  (21.80) | 40 (34.78) | 56 (33.94) |  |  |
|  | Mixed experience | 10  (21.23) | 22 (33.85) | 18  (23.08) | 41 (35.65) | 17 (10.30) |  |  |
| Has current oral HRT been appropriately optimised^a,h^ | Not at all | 7  (14.89) | 6  (9.23) | 10  (12.82) | 26 (22.61) | 17 (10.30) | <0.001*** | UK<CAN,NZ,US |
|  | Yes, partially | 11  (23.40) | 21 (32.31) | 15  (19.23) | 45 (39.13) | 47 (28.49) |  |  |
|  | Yes, mostly | 22  (46.81) | 27 (41.54) | 37  (47.44) | 33 (28.70) | 62 (37.58) |  |  |
|  | Yes, completely | 7  (14.89) | 11 (16.92) | 16  (20.51) | 11  (9.57) | 39 (23.64) |  |  |
| Satisfaction with tolerability of current oral HRT^a,h^ | Extremely dissatisfied | 2  (4.26) | 2  (3.08) | 2  (2.56) | 3  (2.61) | 3  (1.82) | <0.001*** | UK<CAN,NZ,US |
|  | Dissatisfied | 1  (2.13) | 3  (4.62) | 3  (3.85) | 14 (12.17) | 12  (7.27) |  |  |
|  | Neutral | 12  (25.53) | 6  (9.23) | 10  (12.82) | 35 (30.44) | 21 (12.73) |  |  |
|  | Satisfied | 16  (34.04) | 31 (47.69) | 38  (48.72) | 39 (33.91) | 60 (36.36) |  |  |
|  | Extremely satisfied | 16  (24.04) | 23 (35.39) | 25  (32.05) | 24 (20.87) | 69 (41.82) |  |  |
| Overall satisfaction with current oral HRT^a,h^ | Extremely dissatisfied | 2  (4.26) | 2  (3.08) | 1  (1.28) | 2  (1.74) | 0  (0.00) | <0.001*** | UK<CAN,US |
|  | Dissatisfied | 2  (4.26) | 2  (3.08) | 4  (5.13) | 11  (9.57) | 8  (4.85) |  |  |
|  | Neutral | 7  (14.89) | 7  (10.77) | 10  (12.82) | 35  (30.44) | 21  (12.73) |  |  |
|  | Satisfied | 24  (51.06) | 32 (49.23) | 42  (53.85) | 42 (36.52) | 79 (47.88) |  |  |
|  | Extremely satisfied | 12  (25.53) | 22 (33.85) | 21  (26.92) | 25 (21.74) | 57 (34.55) |  |  |
| How long to be prescribed current vaginal HRT by HCP^a,i^ | At first contact | 18 (36.00) | 24 (47.06) | 16 (32.65) | 55 (38.19) | 66 (44.90) | 0.93 | - |
|  | 1 – 3 months | 7 (14.00) | 4 (7.84) | 4 (8.16) | 22 (15.28) | 16 (10.88) |  |  |
|  | 3 – 6 months | 7 (14.00) | 1 (1.96) | 4 (8.16) | 16 (11.11) | 7 (4.76) |  |  |
|  | 6 months – 1 year | 6 (12.00) | 4 (7.84) | 11 (22.45) | 11 (7.64) | 9 (6.12) |  |  |
|  | 1 – 2 years | 4 (8.00) | 4 (7.84) | 7 (14.29) | 14 (9.72) | 19 (12.93) |  |  |
|  | More than 2 years | 8 (16.00) | 14 (27.45) | 7 (14.29) | 26 (18.06) | 30 (20.41) |  |  |
| Number of different HCPs before being prescribed current vaginal HRT^a,j^ | 1 | 12 (37.50) | 9 (33.33) | 11 (33.33) | 27 (30.34) | 24 (29.63) | 0.64 | - |
|  | 2 – 3 | 14 (43.75) | 7 (25.93) | 16 (48.49) | 38 (42.70) | 38 (46.91) |  |  |
|  | 3 – 4 | 5 (15.63) | 6 (22.22) | 3 (9.09) | 11 (12.36) | 8 (9.88) |  |  |
|  | 4 – 5 | 1 (3.13) | 1 (3.70) | 0 (0.00) | 6 (6.74) | 7 (8.64) |  |  |
|  | More than 5 | 0 (0.00) | 4 (14.82) | 3 (9.09) | 7 (7.87) | 4 (4.94) |  |  |
| Ease of being prescribed current vaginal HRT by HCP^a,i^ | Very difficult | 1 (2.00) | 3 (5.88) | 3 (6.12) | 6 (4.17) | 11 (7.48) | 0.92 | - |
|  | Difficult | 8 (16.00) | 8 (15.69) | 7 (14.29) | 25 (17.36) | 21 (14.29) |  |  |
|  | Neutral | 6 (12.00) | 7 (13.73) | 6 (12.25) | 22 (15.28) | 15 (10.20) |  |  |
|  | Easy | 16 (32.00) | 11 (21.57) | 19 (38.78) | 38 (26.39) | 45 (30.61) |  |  |
|  | Very easy | 19 (38.00) | 22 (43.14) | 14 (28.57) | 53 (36.81) | 55 (37.42) |  |  |
| Length of current vaginal HRT use^a,i^ | Less than 3 months | 11 (22.00) | 13 (25.49) | 12 (24.49) | 21 (14.58) | 37 (25.17) | 0.68 | - |
|  | 3 – 6 months | 6 (12.00) | 5 (9.80) | 9 (18.37) | 24 (16.67) | 22 (14.97) |  |  |
|  | 6 months – 1 year | 10 (20.00) | 11 (21.57) | 6 (12.25) | 34 (23.67) | 30 (20.41) |  |  |
|  | 1 – 2 years | 11 (22.00) | 11 (21.57) | 11 (22.45) | 32 (23.61) | 25 (17.01) |  |  |
|  | More than 2 years | 12 (24.00) | 11 (21.57) | 11 (22.45) | 33 (22.92) | 33 (22.45) |  |  |
| Extent to which involved in discussing current vaginal HRT use with HCP^a,k^ | Not at all | 3 (7.69) | 5 (13.16) | 13 (35.14) | 48 (39.02) | 30 (27.27) | 0.41 | - |
|  | To a small extent | 8 (20.51) | 11 (28.95) | 4 (10.81) | 18 (14.63) | 23 (20.91) |  |  |
|  | To some extent | 5 (12.82) | 8 (21.05) | 9 (24.32) | 20 (16.26) | 27 (24.55) |  |  |
|  | To a moderate extent | 11 (28.21) | 9 (23.68) | 9 (24.32) | 19 (15.45) | 21 (19.09) |  |  |
|  | To a large extent | 12 (30.77) | 5 (13.16) | 2 (5.41) | 18 (14.63) | 9 (8.18) |  |  |
| Has current vaginal HRT been appropriately reviewed by HCP^a,k^ | Yes | 25 (64.10) | 18 (47.37) | 20 (54.05) | 54 (43.90) | 64 (58.18) | 0.01* | UK<US |
|  | No | 7 (17.95) | 6 (15.79) | 8 (21.62) | 43 (34.96) | 12 (10.91) |  |  |
|  | Mixed experience | 7 (17.95) | 14 (36.84) | 9 (24.32) | 26 (21.24) | 34 (30.91) |  |  |
| Has current vaginal HRT been appropriately optimised^a,k^ | Not at all | 5 (12.82) | 6 (15.79) | 4 (10.81) | 21 (17.07) | 8 (7.27) | 0.27 | - |
|  | Yes, partially | 6 (15.39) | 9 (23.68) | 18 (48.65) | 30 (24.39) | 31 (28.18) |  |  |
|  | Yes, mostly | 15 (38.46) | 18 (47.37) | 7 (18.92) | 40 (32.52) | 45 (40.91) |  |  |
|  | Yes, completely | 13 (33.33) | 6 (15.79) | 8 (21.62) | 32 (26.02) | 26 (23.64) |  |  |
| Satisfaction with tolerability of current vaginal HRT^a,k^ | Extremely dissatisfied | 1 (2.56) | 1 (2.63) | 0 (0.00) | 2 (1.63) | 1 (0.91) | 0.02* | NZ<US |
|  | Dissatisfied | 4 (10.26) | 2 (5.26) | 6 (16.22) | 12 (9.76) | 3 (2.73) |  |  |
|  | Neutral | 4 (10.26) | 8 (21.05) | 8 (21.62) | 21 (17.07) | 15 (13.64) |  |  |
|  | Satisfied | 20 (51.28) | 11 (28.95) | 17 (45.95) | 46 (37.40) | 45 (40.91) |  |  |
|  | Extremely satisfied | 10 (25.64) | 16 (42.11) | 6 (16.22) | 42 (34.15) | 46 (41.82) |  |  |
| Overall satisfaction with current vaginal HRT^a,k^ | Extremely dissatisfied | 1 (2.56) | 0 (0.00) | 0 (0.00) | 2 (1.63) | 2 (2.73) | 0.26 | - |
|  | Dissatisfied | 4 (10.26) | 3 (7.90) | 3 (8.11) | 8 (6.50) | 5 (4.55) |  |  |
|  | Neutral | 4 (10.26) | 6 (15.79) | 14 (37.84) | 24 (19.51) | 21 (19.09) |  |  |
|  | Satisfied | 20 (51.28) | 17 (44.74) | 14 (37.84) | 55 (44.72) | 50 (45.46) |  |  |
|  | Extremely satisfied | 10 (25.64) | 12 (31.58) | 6 (16.22) | 34 (27.64) | 31 (28.18) |  |  |
| Current antidepressant use^l^ | I was taking antidepressants before the menopause and have continued taking them in the menopause (without a break) | 29 (55.77) | 34 (53.13) | 49 (58.33) | 68 (63.55) | 73 (58.40) | 0.90 | - |
|  | I have taken antidepressants in the past and was advised to start taking them again now I am in the menopause | 9 (17.31) | 16 (25.00) | 15 (21.88) | 19 (17.76) | 24 (19.20) |  |  |
|  | I am currently taking antidepressants for the menopause but have not taken them in the past | 14 (26.92) | 14 (21.88) | 20 (23.81) | 20 (18.69) | 28 (22.40) |  |  |
| How long to be prescribed current antidepressants by HCP^a,m^ | At first contact | 9 (39.13) | 13 (43.33) | 15 (42.86) | 21 (53.85) | 21 (40.39) | 0.90 | - |
|  | 1 – 3 months | 4 (17.39) | 4 (13.33) | 3 (8.57) | 7 (17.95) | 15 (28.85) |  |  |
|  | 3 – 6 months | 5 (21.74) | 2 (6.67) | 4 (11.43) | 1 (2.56) | 3 (5.77) |  |  |
|  | 6 months – 1 year | 1 (4.35) | 4 (13.33) | 3 (8.57) | 1 (2.56) | 5 (9.62) |  |  |
|  | 1 – 2 years | 3 (13.04) | 3 (10.00) | 5 (14.29) | 3 (7.69) | 5 (9.62) |  |  |
|  | More than 2 years | 1 (4.35) | 4 (13.33) | 5 (14.29) | 6 (15.39) | 3 (5.77) |  |  |
| Number of different HCPs before being prescribed current antidepressants HRT^a,n^ | 1 | 6 (42.86) | 10 (58.82) | 7 (35.00) | 4 (22.22) |  | 0.18 | - |
|  | 2 – 3 | 7 (50.00) | 5 (29.41) | 8 (40.00) | 11 (61.11) | 17 (54.84) |  |  |
|  | 3 – 4 | 1 (7.14) | 1 (5.88) | 2 (10.00) | 0 (0.00) | 2 (32.26) |  |  |
|  | 4 – 5 | 0 (0.00) | 0 (0.00) | 3 (15.00) | 3 (16.67) | 2 (6.45) |  |  |
|  | More than 5 | 0 (0.00) | 1 (5.88) | 0 (0.00) | 0 (0.00) | 2 (6.45) |  |  |
| Ease of being prescribed current antidepressants by HCP^a,m^ | Very difficult | 0 (0.00) | 0 (0.00) | 2 (5.71) | 1 (2.56) | 0 (0.00) | 0.03* | NZ<US |
|  | Difficult | 4 (17.39) | 4 (13.33) | 3 (8.57) | 4 (10.26) | 1 (1.92) |  |  |
|  | Neutral | 3 (13.04) | 6 (20.00) | 6 (17.14) | 4 (10.26) | 5 (9.62) |  |  |
|  | Easy | 8 (34.78) | 9 (30.00) | 13 (37.14) | 15 (38.46) | 16 (30.77) |  |  |
|  | Very easy | 8 (34.78) | 11 (36.67) | 11 (31.43) | 15 (38.46) | 30 (57.69) |  |  |
| Length of current antidepressant use^a,l^ | Less than 3 months | 14 (26.92) | 15 (23.44) | 10 (11.91) | 20 (18.69) | 17 (13.60) | 0.08 | - |
|  | 3 – 6 months | 2 (3.85) | 6 (9.38) | 4 (4.76) | 4 (15.89) | 15 (12.00) |  |  |
|  | 6 months – 1 year | 6 (11.54) | 11 (17.19) | 9 (20.71) | 17 (13.08) | 23 (18.40) |  |  |
|  | 1 – 2 years | 12 (23.08) | 10 (15.63) | 20 (23.81) | 14 (13.08) | 18 (14.40) |  |  |
|  | More than 2 years | 18 (34.62) | 22 (34.38) | 41 (48.81) | 52 (48.60) | 52 (41.60) |  |  |
| Extent to which involved in discussing current antidepressant use with HCP^a,o^ | Not at all | 7 (18.42) | 10 (20.41) | 11 (14.87) | 21 (24.14) | 23 (21.30) | 0.08 | - |
|  | To a small extent | 6 (15.79) | 8 (16.33) | 12 (16.22) | 4 (26.44) | 17 (15.74) |  |  |
|  | To some extent | 6 (15.79) | 16 (32.65) | 14 (18.92) | 16 (18.39) | 22 (20.37) |  |  |
|  | To a moderate extent | 8 (21.05) | 5 (10.20) | 19 (25.68) | 15 (17.24) | 21 (19.44) |  |  |
|  | To a large extent | 11 (28.95) | 10 (20.41) | 18 (24.32) | 12 (13.79) | 25 (23.15) |  |  |
| Has current antidepressant use been appropriately reviewed by HCP^a,o^ | Yes | 23 (60.53) | 24 (48.98) | 34 (45.95) | 24 (27.59) | 57 (52.79) | <0.001*** | UK<all others |
|  | No | 8 (21.05) | 12 (26.53) | 21 (28.38) | 44 (50.58) | 19 (17.59) |  |  |
|  | Mixed experience | 7 (18.42) | 13 (26.53) | 19 (25.68) | 19 (21.84) | 32 (29.63) |  |  |
| Has current antidepressant use been appropriately optimised^a,o^ | Not at all | 6 (15.79) | 10 (20.41) | 17 (22.97) | 26 (29.89) | 26 (24.07) | 0.08 | - |
|  | Yes, partially | 11 (28.95) | 17 (34.69) | 15 (20.27) | 30 (34.48) | 26 (24.07) |  |  |
|  | Yes, mostly | 14 (36.84) | 18 (36.74) | 28 (37.84) | 22 (25.29) | 34 (31.48) |  |  |
|  | Yes, completely | 7 (18.42) | 4 (8.16) | 14 (18.92) | 9 (10.35) | 22 (20.37) |  |  |
| Satisfaction with tolerability of current antidepressants^a,o^ | Extremely dissatisfied | 2 (5.26) | 2 (4.08) | 1 (1.35) | 1 (1.15) | 5 (4.63) | 0.14 | - |
|  | Dissatisfied | 7 (18.42) | 4 (8.16) | 5 (6.76) | 9 (20.35) | 6 (5.56) |  |  |
|  | Neutral | 3 (7.90) | 13 (26.53) | 15 (20.27) | 31 (25.63) | 23 (21.30) |  |  |
|  | Satisfied | 13 (34.21) | 15 (30.61) | 32 (43.27) | 30 (34.48) | 38 (35.19) |  |  |
|  | Extremely satisfied | 13 (34.21) | 15 (30.61) | 21 (28.38) | 16 (18.39) | 36 (33.33) |  |  |
| Overall satisfaction with current antidepressants^a,o^ | Extremely dissatisfied | 2 (5.26) | 1 (2.04) | 1 (1.35) | 6 (6.90) | 3 (2.78) | 0.03* | NZ>UK |
|  | Dissatisfied | 4 (10.53) | 10 (20.41) | 5 (6.76) | 8 (9.20) | 12 (11.11) |  |  |
|  | Neutral | 8 (21.05) | 8 (16.33) | 15 (20.27) | 30 (34.48) | 25 (23.15) |  |  |
|  | Satisfied | 16 (42.11) | 21 (42.86) | 31 (41.89) | 31 (35.63) | 41 (37.96) |  |  |
|  | Extremely satisfied | 8 (21.05) | 9 (18.37) | 22 (29.73) | 12 (13.79) | 27 (25.00) |  |  |
| Current testosterone use^p^ | I was taking testosterone before the menopause and have continued to take it in the menopause (without a break) | 0 (0.00) | 0 (0.00) | 0 (0.00) | 0 (0.00) | 2 (2.00) | 0.07 | - |
|  | I have taken testosterone in the past and was advised to take it again now I am in the menopause | 0 (0.00) | 0 (0.00) | 2 (15.39) | 0 (0.00) | 6 (6.00) |  |  |
|  | I am currently taking testosterone for the menopause but have not taken it in the past | 14 (100.00) | 18 (10.00) | 11 (84.62) | 81 (100.00) | 92 (92.00) |  |  |
| How long to be prescribed current testosterone by HCP^a,q^ | At first contact | 4 (28.57) | 2 (11.11) | 2 (15.39) | 18 (22.22) | 26 (26.53) | 0.85 | - |
|  | 1 – 3 months | 3 (21.43) | 2 (11.11) | 2 (15.39) | 7 (8.64) | 15 (15.31) |  |  |
|  | 3 – 6 months | 0 (0.00) | 3 (16.67) | 3 (23.08) | 9 (11.11) | 9 (9.18) |  |  |
|  | 6 months – 1 year | 2 (14.29) | 5 (27.78) | 1 (7.69) | 16 (19.75) | 12 (12.25) |  |  |
|  | 1 – 2 years | 2 (14.29) | 3 (16.67) | 1 (7.69) | 13 (16.05) | 15 (15.31) |  |  |
|  | More than 2 years | 3 (21.43) | 3 (16.67) | 4 (30.77) | 18 (22.22) | 21 (21.43) |  |  |
| Number of different HCPs before being prescribed current testosterone^a,r^ | 1 | 2 (20.00) | 3 (18.75) | 4 (36.26) | 11 (17.46) | 20 (27.78) | 0.91 | - |
|  | 2 – 3 | 5 (50.00) | 9 (56.25) | 4 (36.34) | 36 (57.14) | 34 (47.22) |  |  |
|  | 3 – 4 | 2 (20.00) | 2 (12.50) | 1 (9.09) | 6 (9.52) | 5 (6.94) |  |  |
|  | 4 – 5 | 1 (10.00) | 1 (6.25) | 1 (9.09) | 5 (7.94) | 8 (11.11) |  |  |
|  | More than 5 | 0 (0.00) | 1 (6.25) | 1 (9.09) | 5 (7.94) | 5 (6.94) |  |  |
| Ease of being prescribed current testosterone by HCP^a,q^ | Very difficult | 2 (14.29) | 5 (27.78) | 2 (15.39) | 16 (19.75) | 12 (12.25) | 0.10 | - |
|  | Difficult | 5 (35.71) | 3 (16.67) | 4 (30.77) | 17 (20.99) | 16 (16.33) |  |  |
|  | Neutral | 3 (21.43) | 3 (16.67) | 3 (23.08) | 9 (11.11) | 14 (14.29) |  |  |
|  | Easy | 3 (21.43) | 4 (22.22) | 1 (7.69) | 24 (29.63) | 26 (26.53) |  |  |
|  | Very easy | 1 (7.14) | 3 (16.67) | 3 (23.08) | 15 (18.52) | 30 (30.61) |  |  |
| Length of current testosterone use^a,p^ | Less than 3 months | 4 (28.57) | 7 (38/89) | 3 (23.08) | 12 (14.82) | 20 (20.00) | 0.05* | NS |
|  | 3 – 6 months | 4 (28.57) | 3 (16.67) | 1 (7.69) | 10 (12.35) | 23 (23.00) |  |  |
|  | 6 months – 1 year | 3 (21.43) | 2 (11.11) | 5 (38.46) | 12 (14.82) | 27 (27.00) |  |  |
|  | 1 – 2 years | 1 (7.14) | 1 (5.56) | 2 (15.39) | 29 (35.80) | 11 (11.00) |  |  |
|  | More than 2 years | 2 (14.29) | 5 (27.78) | 2 (15.39) | 18 (22.22) | 0 (0.00) |  |  |
| Extent to which involved in discussing current testosterone with HCP^a,s^ | Not at all | 0 (0.00) | 0 (0.00) | 0 (0.00) | 6 (8.70) | 0 (0.00) | 0.09 | - |
|  | To a small extent | 1 (10.00) | 1 (9.09) | 1 (10.00) | 9 (13.04) | 7 (8.75) |  |  |
|  | To some extent | 3 (30.00) | 1 (9.09) | 2 (20.00) | 8 (11.59) | 13 (16.25) |  |  |
|  | To a moderate extent | 1 (10.00) | 1 (9.09) | 1 (10.00) | 20 (28.99) | 14 (17.50) |  |  |
|  | To a large extent | 5 (50.00) | 8 (72.73) | 6 (60.00) | 26 (37.68) | 46 (57.50) |  |  |
| Has current testosterone been appropriately reviewed by HCP^a,s^ | Yes | 7 (70.00) | 8 (72.73) | 6 (60.00) | 34 (49.28) | 58 (72.50) | 0.02* | UK<US |
|  | No | 1 (10.00) | 0 (0.00) | 3 (30.00) | 15 (21.74) | 4 (5.00) |  |  |
|  | Mixed experience | 2 (20.00) | 3 (27.27) | 1 (10.00) | 20 (28.99) | 18 (22.50) |  |  |
| Has current testosterone been appropriately optimised^a,s^ | Not at all | 1 (10.00) | 0 (0.00) | 1 (10.00) | 9 (13.04) | 6 (7.50) | 0.40 | - |
|  | Yes, partially | 4 (40.00) | 3 (27.27) | 2 (20.00) | 20 (28.99) | 23 (28.75) |  |  |
|  | Yes, mostly | 4 (40.00) | 4 (36.36) | 4 (40.00) | 28 (40.58) | 31 (38.75) |  |  |
|  | Yes, completely | 1 (10.00) | 4 (36.36) | 3 (30.00) | 12 (17.39) | 20 (25.00) |  |  |
| Satisfaction with tolerability of current testosterone^a,s^ | Extremely dissatisfied | 0 (0.00) | 0 (0.00) | 1 (10.00) | 1 (1.49) | 0 (0.00) | 0.73 | - |
|  | Dissatisfied | 0 (0.00) | 0 (0.00) | 1 (10.00) | 1 (1.49) | 3 (3.75) |  |  |
|  | Neutral | 2 (20.00) | 1 (9.09) | 3 (30.00) | 12 (17.39) | 17 (21.25) |  |  |
|  | Satisfied | 5 (50.00) | 5 (45.46) | 1 (10.00) | 32 (46.38) | 31 (38.75) |  |  |
|  | Extremely satisfied | 3 (30.00) | 5 (45.46) | 4 (40.00) | 23 (33.33) | 29 (36.25) |  |  |
| Overall satisfaction with current testosterone^a,s^ | Extremely dissatisfied | 0 (0.00) | 0 (0.00) | 1 (10.00) | 0 (0.00) | 0 (0.00) | 0.92 | - |
|  | Dissatisfied | 0 (0.00) | 0 (0.00) | 0 (0.00) | 2 (2.90) | 3 (3.75) |  |  |
|  | Neutral | 3 (30.00) | 2 (18.18) | 2 (20.00) | 7 (10.15) | 18 (22.50) |  |  |
|  | Satisfied | 4 (40.00) | 5 (45.46) | 3 (30.00) | 40 (57.97) | 34 (42.50) |  |  |
|  | Extremely satisfied | 3 (30.00) | 4 (36.36) | 4 (40.00) | 20 (28.99) | 25 (31.25) |  |  |
| Length of current CBT/therapy/counselling^a,u^ | Less than 3 months | 6 (23.08) | 12 (33.33) | 3 (13.64) | 19 (61.29) | 19 (20.88) | <0.001*** | AUS,NZ,US>UK |
|  | 3 – 6 months | 4 (15.39) | 6 (16.67) | 6 (27.27) | 7 (22.58) | 8 (8.79) |  |  |
|  | 6 months – 1 year | 6 (23.08) | 6 (16.67) | 1 (4.55) | 1 (22.58) | 17 (18.68) |  |  |
|  | 1 – 2 years | 5 (19.23) | 4 (11.11) | 3 (13.64) | 2 (6.45) | 18 (19.78) |  |  |
|  | More than 2 years | 5 (19.23) | 8 (22.22) | 9 (40.91) | 2 (6.45) | 0 (0.00) |  |  |
| Overall satisfaction with current CBT/therapy/counselling^a,v^ | Extremely dissatisfied | 1 (5.00) | 0 (0.00) | 0 (0.00) | 0 (0.00) | 4 (5.56) | 0.38 | - |
|  | Dissatisfied | 0 (0.00) | 1 (4.35) | 0 (0.00) | 0 (0.00) | 2 (2.78) |  |  |
|  | Neutral | 5 (25.00) | 2 (8.70) | 4 (21.05) | 5 (41.67) | 11 (15.28) |  |  |
|  | Satisfied | 22 (55.00) | 11 (47.83) | 10 (52.63) | 4 (33.33) | 30 (41.67) |  |  |
|  | Extremely satisfied | 3 (15.00) | 9 (39.13) | 5 (26.32) | 3 (25.00) | 25 (34.72) |  |  |

***Note.*** AUS, Australia; CAN, Canada; CBT, cognitive behavioural therapy; HCP, health care professional; HRT, hormone replacement therapy; NS, non-significant (i.e., did not survive Bonferroni correction for multiple comparisons); NZ, New Zealand; UK, United Kingdom; US, United States.

***Key.*** **p*<0.05; ***p*<0.01; ****p*<0.001
^a^ data treated as ordinal
^b^ includes those currently using transdermal HRT (*N*=1025)
^c^ includes those who were *not* prescribed their current transdermal HRT at first contact (*N*=640)
^d^ includes those currently using transdermal HRT for longer than 3 months (*N*=871)
^e^ missing data from 2 respondents as they had initially selected ‘past use’ but open-ended responses suggested ongoing use
^f^ includes those currently taking oral HRT (*N*=562)
^g^ includes those who were *not* prescribed their current oral HRT at first contact (*N*=353)
^h^ includes those currently using oral HRT from longer than 3 months (*N*=470)
^i^ includes those currently using vaginal HRT (*N*=441)
^j^ includes those who were *not* prescribed their current vaginal HRT at first contact (*N*=262)
^k^ includes those currently using vaginal HRT for longer than 3 months (*N*=347)
^l^ includes those currently taking antidepressants (*N*=432)
^m^ includes those who had taken antidepressants in the past and are currently taking them again (with a break) and those who had never taken them in the past and are now taking them (*N*=179)
^n^ includes those who were not prescribed their current antidepressants at first contact (*N*=100)
^o^ includes those currently taking antidepressants for longer than 3 months (*N*=356)
^p^ includes those currently using testosterone (*N*=226)
^q^ includes those who had used testosterone in the past and are currently using it again (with a break) and those who had never used it in the past and are now using it (*N*=224)
^r^ includes those who were not prescribed their current testosterone at first contact (*N*=172)
^s^ includes those currently using testosterone for longer than 3 months (*N*=180)
^t^ missing data from 4 respondents as they had initially selected ‘past use’ but open-ended responses suggested ongoing use
^u^ includes those currently using CBT/therapy/counselling (*N*=206)
^v^ includes those currently using CBT/therapy/counselling for longer than 3 months (*N*=146; data missing from 1 respondent)
